# Influence of Ultrasonication of Functionalized Carbon Nanotubes on the Rheology, Hydration, and Compressive Strength of Portland Cement Pastes

**DOI:** 10.3390/ma14185248

**Published:** 2021-09-13

**Authors:** Laura Silvestro, Artur Ruviaro, Geannina Lima, Paulo de Matos, Afonso R. G. de Azevedo, Sérgio Neves Monteiro, Philippe Gleize

**Affiliations:** 1 Department of Civil Engineering, Federal University of Santa Catarina (UFSC), Florianópolis 88040-900, Brazil; laura.silvestro@posgrad.ufsc.br (L.S.); artur.spat@posgrad.ufsc.br (A.R.); geannina.lima@posgrad.ufsc.br (G.L.); p.gleize@ufsc.br (P.G.); 2 Coordenadoria Acadêmica, Federal University of Santa Maria (UFSM), Cachoeira do Sul 96503-205, Brazil; paulorm.matos@gmail.com; 3 LECIV—Civil Engineering Laboratory, UENF—State University of the Northern Rio de Janeiro, Av. Alberto Lamego, 2000, Campos dos Goytacazes 28013-602, Brazil; 4 Military Engineering Institute, IME—Materials Science Program, Praça Gen. Tibúrcio, 80, Urca, Rio de Janeiro 22290-270, Brazil; snevesmonteiro@gmail.com

**Keywords:** carbon nanotube, ultrasonication, hydration, rheology, compressive strength

## Abstract

The functionalization process usually increases the localized defects of carbon nanotubes (CNT). Thus, the ultrasonication parameters used for dispersing non-functionalized CNT should be carefully evaluated to verify if they are adequate in dispersing functionalized CNT. Although ultrasonication is widely used for non-functionalized CNT, the effect of this dispersing process of functionalized CNT has not been thoroughly investigated. Thus, this work investigated the effect of ultrasonication on functionalized CNT + superplasticizer (SP) aqueous dispersions by ultraviolet-visible (UV-Vis) spectroscopy, dynamic light scattering (DLS), and Fourier transform infrared spectroscopy (FTIR). Furthermore, Portland cement pastes with additions of 0.05% and 0.1% CNT by cement weight and ultrasonication amplitudes of 0%, 50% and 80% were evaluated through rheometry, isothermal calorimetry, compressive strength at 1, 7 and 28 days, X-ray diffraction (XRD), and thermogravimetric analysis (TGA). FTIR results from CNT + SP dispersions indicated that ultrasonication may negatively affect SP molecules and CNT graphene structure. The increase in CNT content and amplitude of ultrasonication gradually increased the static and dynamic yield stress of paste but did not significantly affect its hydration kinetics. Compressive strength results indicated that the optimum CNT content was 0.05% by cement weight, which increased the strength of composite by up to 15.8% compared with the plain paste. CNT ultrasonication neither increases the degree of hydration of cement nor the mechanical performance of composite when compared with mixes containing unsonicated CNT. Overall, ultrasonication of functionalized CNT is not efficient in improving the fresh and hardened performance of cementitious composites.

## 1. Introduction

The use of carbon nanotubes (CNT) is still limited due to difficulties associated with the dispersion of these nanomaterials and their weak interaction with cementitious matrix. This is because CNT, unlike other spherical particles and carbon fibers, are characterized by small nanometric diameters, high aspect ratio (>1000), and, therefore, a large surface area [1,2]. The effect of CNT on the mechanical performance of cement-based materials is not a consensus in the literature. [3,4]. Some authors have reported that CNT significantly increased the mechanical performance of cementitious composites. By contrast, other authors have observed decreases in these properties [4]. Mohsen et al. [3] attribute these divergences to inefficient CNT dispersion.

CNT dispersion can be carried out using physical and chemical methods. Ultrasonication, ball milling, magnetic stirring, among others can be classified as physical methods. Furthermore, according to the bond type that occurs on the CNT surface, the chemical methods can be subdivided into covalent and non-covalent functionalization [5]. According to Liew et al. [5], the most commonly used methods in experimental research to disperse CNT in water are ultrasonication and dispersant agents. 

According to a survey carried out by Silvestro and Gleize [6] which evaluated 99 works related to use of CNT in cementitious matrix, most works used ultrasonication and dispersant agents to disperse the nanomaterial. Polycarboxylate-based admixtures are the most used type, identified in 40% of those works. Besides CNT dispersion, polycarboxylate-based admixtures are also used to ensure the workability of cement mixes.

The use of dispersant agents for CNT dispersion can be classified as a non-covalent functionalization process, with the main feature of not modifying the original structure of CNT. Banerjee et al. [7] reported that this type of functionalization avoid changes in the sp^2^ CNT structure.

The ultrasonication process aims to disperse nanomaterials through the application of ultrasound energy. The dispersion of CNT agglomerates occurs due to the implosion of microbubbles. There are two types of equipment used in ultrasonication, named ultrasound bath and ultrasound tip. In this context, the ultrasound tip promotes better dispersion of the CNT, as it provides a denser amount of energy [5]. 

The energy applied in the CNT dispersion process must be carefully evaluated, considering that if it is excessive it can cause the breakage of CNT and, thus, reduce the efficiency of incorporation of the nanomaterial on the mechanical performance of cement-based materials [8]. Jarolim et al. [8] found the value of 800 J/mL to be the optimal amount of ultrasonic energy for CNT dispersion through UV-Vis spectroscopy. This energy resulted in a well-dispersed CNT dispersion, as observed by optical microscopy, and increased the compressive and flexural strengths at 7 and 28 days of curing mortars. However, it is worth noting that alkaline environments, such as cement pore solution, affect the stability of aqueous CNT dispersions [9]. Thus, the dispersion of CNT in aqueous solution can change when in contact with cement particles. Therefore, the behavior of CNT in aqueous solutions cannot be extrapolated for CNT dispersion in cementitious composites. 

According to Zou et al. [10], the optimal ultrasonication energy balances the degree of dispersion and the shortening of CNT agglomerates to achieve superior mechanical properties. Regarding the dispersion in water assessed by UV-Vis spectroscopy, Zou et al. [10] observed that the absorbance, directly related to the degree of dispersion of the CNT aqueous solution, gradually increases with increasing ultrasonication energy until reaching a plateau between 150 and 250 J/mL. In turn, considering the modulus of elasticity and flexural strength of cement pastes, the authors obtained the best mechanical performance with the energy ultrasonication value of 20 J/mL. The results obtained by these authors indicated that the optimum energies for aqueous dispersions and for cementitious composites were not the same. This reinforces the fragility of the extrapolation of behaviors observed in CNT aqueous solution for CNT dispersion in cementitious matrix. 

Regarding the influence of the ultrasonication time, Isfahani, et al. [11] characterized the aqueous dispersions of non-functionalized CNT and COOH-CNT through UV-Vis spectroscopy. The CNT were dispersed by ultrasonication for 0, 30, 60 and 120 min. According to their results of UV-Vis spectroscopy, the best dispersion in water was obtained after 120 min for functionalized CNT. For non-functionalized CNT, it was obtained after 60 min. No significant increases in the compressive and flexural strengths of mortars containing the two types of CNT were observed with longer ultrasonication times. These authors also indicated that the high dispersion of CNT in cementitious matrix was not obtained by using a high CNT dispersion. 

Siqueira and Gleize [12] analyzed the influence of amplitude (20%, 50% and 80%) and ultrasonication time (6, 30 and 60 min) on the dispersion of aqueous non-functionalized CNT dispersions and on mechanical properties of cementitious composites. The authors observed that the use of high energy combined with the short ultrasonication time (amplitude of 80% and duration of 6 min) generated the best mechanical performance when compared with the reference (i.e., plain cement paste). With the increase of ultrasonication time, there was a clear reduction in compressive and flexural strength for all amplitudes, which is probably a consequence of the CNT damage. In this context, Alrekabi et al. [13] also mentioned that ultrasonication with high intensity (i.e., amplitude) for short periods leads to a better CNT dispersion and, therefore, to a greater efficiency in the mechanical reinforcement of cementitious composites.

It is also worth mentioning the lack of information regarding the parameters used in the CNT dispersion process via ultrasonication. This makes direct comparison of results complex and limits the reproduction of previously published studies [6,12]. Additionally, with the exception of the work from Isfahani et al. [11], other previously mentioned works [10,12,13] evaluating the influence of ultrasonication energy on the mechanical properties of cementitious composites did not compare the effect of different ultrasonication times and amplitudes with composites with CNT that were not dispersed by ultrasonication. Furthermore, although some works [10,11,12,13] have evaluated the influence of CNT ultrasonication energy on the mechanical properties of cementitious composites, the effect of this dispersion process on aqueous CNT + SP solutions has not been thoroughly investigated. In that context, Assi et al. [14] mentioned that the ultrasonication of water molecules produce hydrogen peroxide (H_2_O_2_), which reacts with the calcium hydroxide [Ca(OH)_2_]. The product formed by this reaction is calcium peroxide (CaO_2_), a salt that can improve the compressive strength gain at early ages [14]. However, the effect of ultrasonication of superplasticizer (SP) molecules has not yet been reported.

Considering this gap in knowledge, the aim of this work is to elucidate the influence of the ultrasonication process on rheology, hydration kinetics, compressive strength, and microstructure of cementitious composites with functionalized CNT without ultrasonication and pastes with CNT that were previously ultrasonicated with two amplitudes (50% and 80%) [12]. For this purpose, CNT dispersions were characterized by UV-Vis spectroscopy, dynamic light scattering (DLS), and Fourier transform infrared spectroscopy (FTIR). Additionally, rheological tests were conducted though rotational rheometry and the hydration kinetics was evaluated by isothermal calorimetry. Compressive strength of composites was determined at 1, 7 and 28 curing days. The microstructure was evaluated through X-ray diffraction (XRD) and thermogravimetric analysis (TGA).

### Originality

The type of CNT most used in cementitious matrix is non-functionalized, which is usually dispersed by ultrasonication [6,15,16]. In this context, although some works have evaluated the influence of CNT ultrasonication energy on the mechanical properties of cementitious composites, the lack of characterization of the effect of this dispersion process in aqueous CNT + SP solutions is highlighted. 

Furthermore, since the use of functionalized CNT is less usual, the effectiveness of using ultrasonication to disperse functionalized CNT and improve the fresh and hardened performance of cementitious composites has not been fully investigated. Finally, rheological characterizations and hydration kinetics evaluations of CNT-reinforced cement pastes are still relatively scarce and can also be highlighted as a significant contribution from this work.

## 2. Materials and Methods

### 2.1. Materials

**Figure 1:**
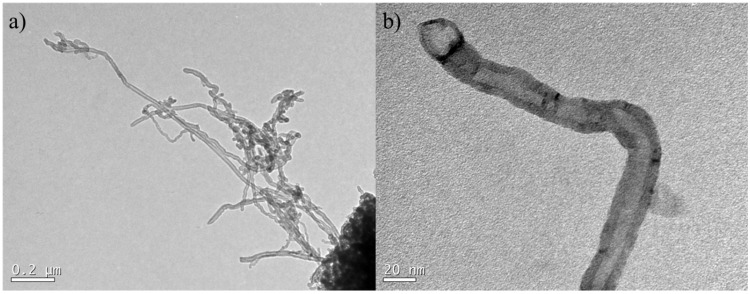
TEM images of the CNT used (**a**) 0.2 µm range; (**b**) 20 nm range.

Carboxyl multi-walled carbon nanotubes (CNT) were used in this experiment. CNT was supplied by Nanostructured & Amorphous Materials Inc., and its base properties are shown in Table 1. The transmission electron microscopy (TEM, JEM-1011, Joel, Akishima, Japan) image of CNT is show in Figure 1. CNT exhibited an average diameter about 20–30 nm, in agreement with the information provided by the manufacturer. Furthermore, TEM image (Figure 1a) shows an agglomeration trend of CNT. The Raman spectroscopy of CNT was conducted in a Renishaw 2000 spectrometer. The results indicated that CNT have an I_D_/I_G_ ratio of 0.98. The disorder band (D) is generated by disorder attributed to sp^3^ hybridized carbon systems, while the graphite (G) band is attributed to sp^2^ bonds. Thus, the I_D_/I_G_ ratio can be used to evaluate the short-range order of the CNT structure [17]. The I_D_/I_G_ ratio of the carboxyl-functionalized CNT used in this study is higher than the I_D_/I_G_ ratio of the non-functionalized CNT reported in previous studies. Batiston et al. [18] used non-functionalized CNT with an I_D_/I_G_ ratio of 0.89. The non-functionalized CNT evaluated by Bogas et al. [19] showed an I_D_/I_G_ ratio of 0.85. These Raman results show that CNT functionalized with the carboxyl groups have a higher amount of localized defects in the sp^2^ network compared to non-functionalized CNT [20]. This suggests that the ultrasonication parameters used for dispersing non-functionalized CNT should be carefully evaluated to verify that they are adequate in dispersing functionalized CNT.

**Figure 2:**
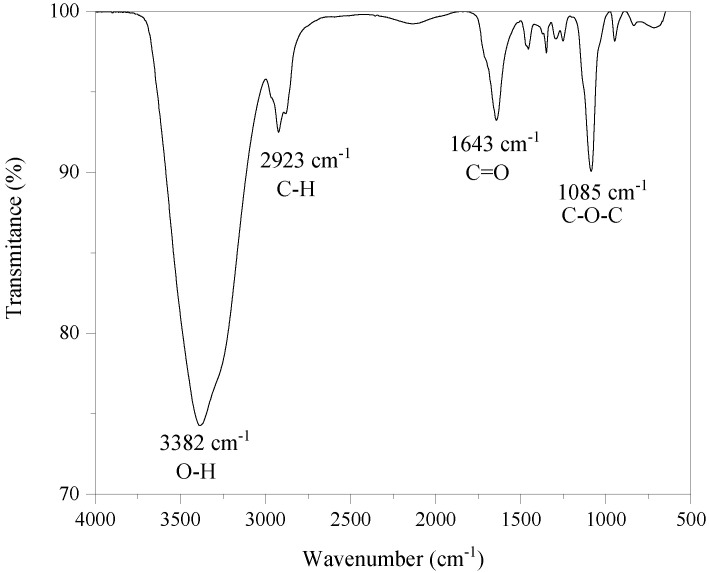
FTIR spectrum of the polycarboxylate-based superplasticizer used.

The admixture used for the dispersion of CNT was the polycarboxylate-based SP MC-PowerFlow 4000 supplied by Mc-Bauchemie. Figure 2 shows the FTIR characterization of the admixture. The test was performed on a liquid sample on a Cary 600 Series FTIR Spectrometer, with an analysis range of 500 to 4000 cm^−1^ and resolution of 2 cm^−1^. Characteristic functional groups of polycarboxylate-based SP were identified, such as spectrum ranges of –OH group, C-H bond of aliphatic organic groups, carbonyl groups (C=O) and ether groups (C–O–C) [21]. This SP admixture has a solid content of 42.1 wt.%.

Ordinary Portland cement was used for paste production. The chemical composition of the cement presented in Table 2 was determined by X-ray fluorescence (XRF) in an EDX-7000 spectrometer (Shimadzu, Tokyo, Japan). The average diameter of Portland cement is 16.53 μm and the density of 3.09 g/cm^3^. The ordinary Portland Cement (OPC) was supplied by Itambé Cimentos (Balsa Nova-PR, Brazil).

### 2.2. Mix Proportions 

Table 3 shows the compositions of the aqueous CNT dispersions evaluated. CNT contents of 0.05% and 0.1% by cement weight were evaluated [6]. The water/cement ratio (w/c) adopted was 0.4 since CNT incorporation is more effective in improving the mechanical strength of cementitious composites with low w/c ratios [22]. The amount of SP was fixed at 0.2% by cement weight. This SP content was incorporated in all evaluated mixtures, including the control paste, i.e., without CNT addition (designated as REF). This value was defined based on the work from Cui et al. [23] since, as verified in UV-Vis spectroscopy, the content of polycarboxylate-based SP admixture that generated the best dispersions of CNT in aqueous solutions was found between 1:2 and 1:4 (NTC:SP, by weight). Furthermore, the SP content was fixed to evaluate the isolated effect of CNT incorporation in the cement pastes. This strategy has already been used by other researchers [24,25]. In addition, the effect of ultrasonication amplitude of CNT on the dispersion and mechanical properties of cementitious composites was studied. For this purpose, CNT solutions without ultrasonication (A0%) and two ultrasonication amplitudes (A50% and A80%) were evaluated. These parameters were defined based on previous works [12,13].

### 2.3. Aqueous CNT Dispersions Production and Characterization

Initially, the appropriate amounts of CNT, deionized water, and SP were weighed, mixed, and ultrasonicated in a probe sonicator Vibra-Cell, VCX Serie, 750 W, 20 KHz, with diameter of 13 mm (Sonics & Materials Inc., Newtown, CT, USA) with an amplitude of 50% or 80% for 6 min. The CNT dispersions were kept in an ice bath during ultrasonication to avoid increases in the temperature. CNT dispersions without ultrasonication were hand mixed for 2 min. 

UV-Vis analyses were performed using a UV-5100S digital spectrophotometer. The CNT aqueous dispersions were diluted in deionized in the proportion of 1:100 [26] with magnetic stirring for 5 min, to keep the absorbance values in the range of 0.1–2 in the entire wavelength range similar to the interval adopted by Attal et al. [27]. In aqueous solutions, the characteristic absorption of CNT occurs at 253 nm [28]. All spectra were obtained as a single scan in 10 mm quartz cuvettes, with a medium scan speed of 0.5 nm intervals, and analysis range from 200 to 600 nm. Quartz cuvettes were used because they are more suitable for the CNT characteristic absorbance range, near to 253 nm. Furthermore, deionized water was used as reference since tests were carried out with aqueous solutions with the same admixture concentration used to CNT dispersion, verifying that the admixture has no influence on the CNT characteristic wavelength (253 nm). Mendoza et al. [9] mentioned that UV-Vis absorption usually depends on the type and degree of substitution of the aromatic center of the molecules. In fact, polycarboxylate-based admixtures, in general, do not contain aromatic groups, it does not interfere in the UV-Vis absorption of CNT aqueous solutions. 

Particle size distribution of CNT aqueous solutions was measured by dynamic light scattering (DLS) in a Zetasizer Nano (Malvern, UK), with a measuring range from 3.8 nm to 100 µm and temperature of 25 °C. After the ultrasonication of CNT, the dispersions were diluted at 1:100 by volume.

FTIR analysis aimed to identify the interaction between CNT and SP. After the ultrasonication of CNT aqueous dispersions, samples were dried for 24 h at 70 °C. The analysis was performed in KBr pellets in a Cary 600 Series FTIR Spectrometer, with an analysis range from 500 to 4000 cm^−1^, resolution of 8 cm^−1^, and 64 accumulations. 

### 2.4. Cementitious Composites Production and Characterization

The CNT aqueous dispersions were added to 100 g of OPC in a solution/cement ratio of 0.40 by weight, and those were mixed for 3 min in a high-shear mixer (10,000 rpm). 

Rheological analyses were performed in a Haake MARS III (Thermo Fisher Scientific, Waltham, MA, USA) rheometer with a four blades vane geometry (Ø16 mm and 22 mm of height). The tests were performed in samples with 25 mL at 23 °C as the procedure described in [29]. The dynamic yield stress ($\tau_{0}$) and equivalent viscosity (µ*_eq_*) were calculated considering the decreasing part of the flow curve using the Herschel-Bulkley model in Equations (1) and (2) [30], respectively.

(1) $$\tau =\tau_{0}+K\cdot {\dot{ɣ}}^{n}$$

(2) $${µ}_{eq}=\frac{3K}{n+2}\cdot {\left({\dot{ɣ}}_{max}\right)}^{n-1}$$

where τ is the shear stress (Pa), $\dot{ɣ}$ is the shear rate (s^−1^), K is the consistency and n is the pseudoplastic parameters, and ${\dot{ɣ}}_{max}$ is the maximum shear rate. In addition, the mini slump test [31] was used to evaluate the flowability of the sample.

The hydration kinetics of cement pastes were evaluated by isothermal calorimetry in a TAM Air (TA Instruments) calorimeter. The test was carried out at a temperature of 23 °C up to 48 h. 

The compressive strength of the CNT cementitious composites was evaluated at 1, 7, and 28 days following ASTM C1231 [32]. Six cylindrical specimens (Ø20 mm × h 26 mm) were cast for each cement paste. Analysis of variance (ANOVA) was conducted to verify whether the CNT content and ultrasonication amplitude evaluated have a significant influence on the compressive strength of cementitious composites. 

X-ray diffraction (XRD) was conducted on a Miniflex II Desktop X-Ray Difractometer (Rigaku, Tokyo, Japan), with 30 kV/15 mA, and CuK radiation (λ = 1.5418 Å). The analysis was performed from 5° to 70° (2θ), and with 0.02° 2θ step size. Thermogravimetric analysis (TGA) was carried out using a SDT Q600 (TA Instruments) at a heating rate of 10 °C/min with a N_2_ flow of 50 mL/min. 

For XRD and TGA analyses, cement hydration was stopped with isopropanol as described in [33]. After that, samples were ground until they passed a 45-µm-opening mesh. 

## 3. Results and Discussion 

### 3.1. Aqueous CNT Dispersions

#### 3.1.1. UV-Vis Spectroscopy

**Figure 3:**
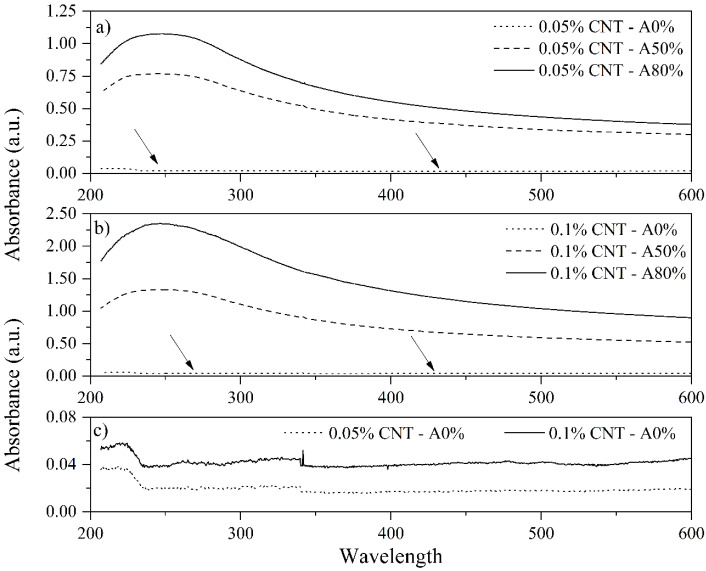
Influence of ultrasonication amplitude on UV-Vis absorption spectra of (**a**) 0.05% CNT; (**b**) 0.1% CNT dispersions and (**c**) amplification of 0.05% CNT—A0% and 0.1% CNT—A0%.

UV-Vis spectroscopy was used to evaluate the quality of CNT dispersions in water. In aqueous solutions with low CNT concentrations, the absorbance obtained by the UV-Vis spectroscopy can be linearly related to the CNT concentration, as described by Lambert-Beer Law [34]. Thus, the higher the absorbance value, the greater the concentration of dispersed CNT. As seen in Figure 3, all the dispersions exhibit a characteristic peak in their UV-Vis spectrum at the wavelength of 253 nm. Furthermore, with increasing ultrasonication amplitude, there was an increase in the CNT dispersion. This behavior is in agreement with previous studies [35,36]. Nevertheless, it is important to consider that ultrasonication may result in two opposite effects, one being the dispersion of CNT clusters and the other the fragmentation of individual CNT. Therefore, UV-Vis alone cannot determine the mean agglomerate size and cannot be used individually to determine the optimum ultrasonication energy for CNT dispersion [37]. Thus, DLS and FTIR tests were performed to complement the characterization of the CNT dispersions.

#### 3.1.2. Particle Size Distribution

**Figure 4:**
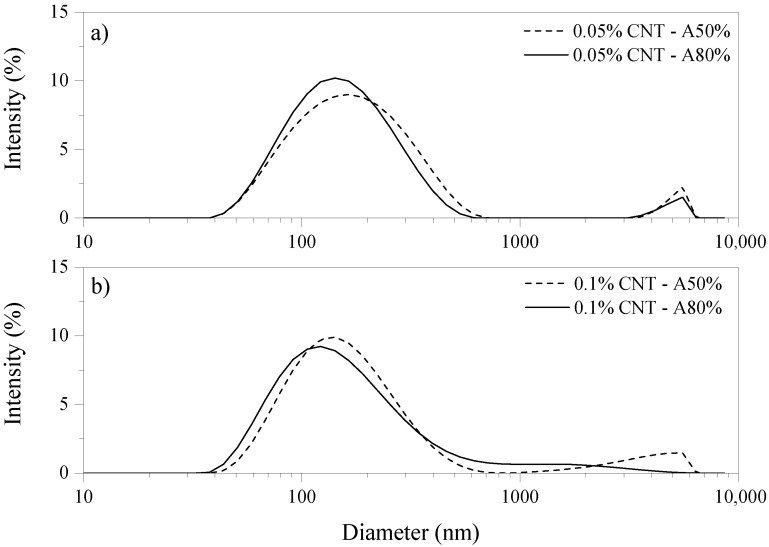
Influence of ultrasonication amplitude on particle size distribution of (**a**) 0.05% CNT and (**b**) 0.1% CNT dispersions.

Figure 4 and Table 4 show the particle size distribution, average particle size, and polydispersity index (PDI) of CNT dispersions. The CNT solutions without ultrasonication were not stable, and it was not possible to perform the dynamic light scattering (DLS) test on these samples. Overall, CNT dispersions showed monomodal distribution. In addition, increasing the amplitude from 50% to 80% did not promote significant changes in the dispersion of CNT measured by DLS. 

PDI is used to evaluate CNT dispersion quality. In general, PDI ˃ 0.5 is associated with poly disperse distributed samples [30]. Thus, ultrasonicated dispersions can be considered homogeneous. It should be stressed that DLS is an accurate technique for measuring the particle size of spherical particles. However, the aspect ratio and CNT agglomerates may affect the particle size results [38]. Reales and Toledo Filho [39] mentioned that the results obtained from this technique are representative of CNT agglomerates. This must be carefully considered when interpreting the results of particle size analysis. According to Chowdhury and Cui [40], the CNT dimensions cannot be directly correlated with the DLS results, since this technique is more suitable for analyzing spherical particles. Nonetheless, it may be used as an indication of agglomeration. 

#### 3.1.3. FTIR

**Figure 5:**
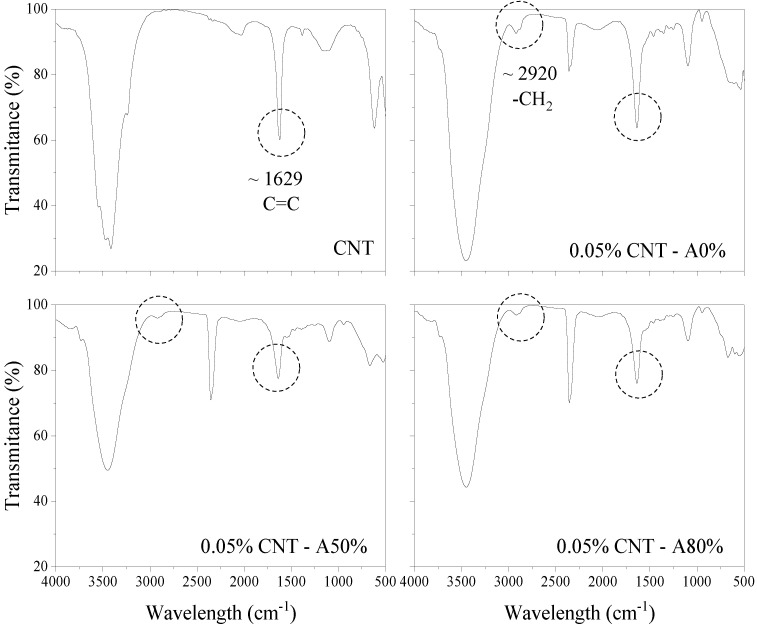
FTIR spectra of polycarboxylate-based superplasticizer and 0.05% CNT dispersions.

**Figure 6:**
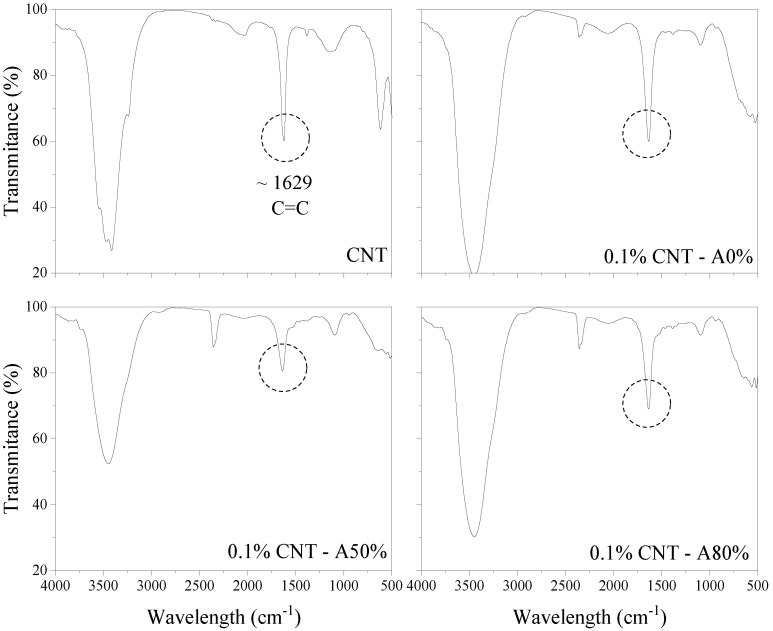
FTIR spectra of polycarboxylate-based superplasticizer and 0.1% CNT dispersions.

The spectra of 0.05% CNT and 0.1% CNT aqueous dispersions are shown in Figure 5 and Figure 6, respectively. New absorption bands in CNT dispersions appeared at 2920 cm^−1^, which are attributed to the stretching vibration of –CH_2_ [41]. This is attributed to SP molecules [41]. The appearance of these bands is more evident in dispersions with 0.05% CNT since they have a higher CNT:SP ratio compared with dispersions with 0.1% CNT. It can also be observed that, in CNT dispersions submitted to ultrasonication, the bands assigned to –CH_2_ were attenuated. This may be an indication that ultrasonication promotes changes in the SP molecules. Similarly, the results obtained by Hu et al. [41] also indicated that the ultrasonication of the polycarboxylate admixture causes an attenuation of the band attributed to –CH_2_. In this context, the studied conducted by Dehghani et al. [42] indicated that the ultrasonication can be used for degradation of surfactants in aqueous solutions. The longer the ultrasonication time and the lower the concentration of surfactant, the greater the degree of degradation. Thus, the effect of ultrasonication on SP admixtures should be further investigated, especially for long periods. It can also be seen in Figure 5 and Figure 6 that, in ultrasonicated CNT dispersions, there was a reduction in the band at 1629 cm^−1^, attributed to the C=C stretching of CNT graphene structure [43,44,45,46,47,48,49]. These results may indicate that the ultrasonication have caused damage to the CNT structure [50]. Previous studies also indicated that the ultrasonication can damage the CNTs [2,12]. 

### 3.2. Cementitious Composite Characterization

#### 3.2.1. Rheological Tests 

**Figure 7:**
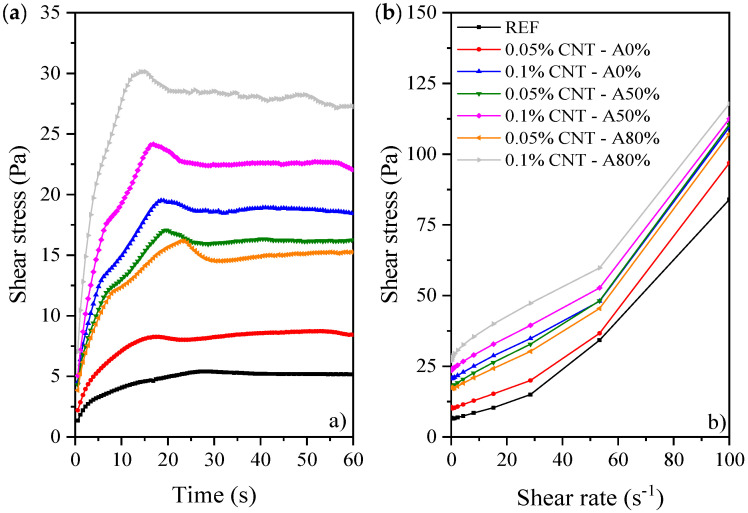
Curves of rheological tests: (**a**) static yield stress determination, and (**b**) flow curves of cement pastes.

**Figure 8:**
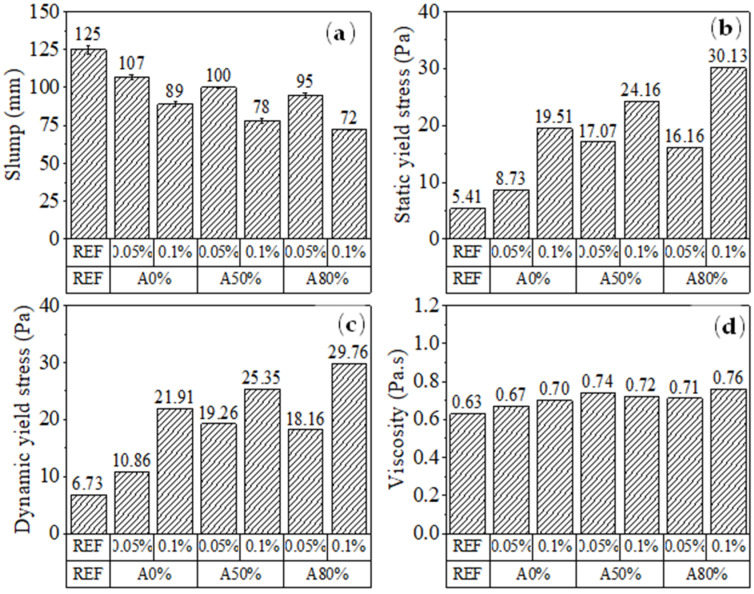
Rheological parameters of CNT cementitious composites: (**a**) slump; (**b**) static and (**c**) dynamic yield stresses, and (**d**) viscosity.

Figure 7 and Figure 8 show the rheological results of cement pastes. The increase of CNT content and amplitude of ultrasonication gradually increased the static and dynamic yield stress and decreased the mini slump of the pastes. The addition of nanomaterials changes the rheological properties of cement pastes and severely reduces their workability. According to Jiang et al. [51], the high specific surface area of CNT is the main reason affecting the rheology of cement pastes. This is because the large surface area of nanomaterial demands more water to wet their surface, consequently reducing the free water available for lubrication at a given w/c ratio [52]. Besides that, CNT can interact with SP admixtures, facilitating their dispersion and reducing the amounts of SP available to interact with the cement grains [53]. This trend may contribute to the increase in viscosity and yield stress of CNT cementitious composites compared with plain cement paste.

Regarding the effect of the ultrasonication amplitude, a higher ultrasonication energy promoted a better CNT dispersion (Seen in Section 3.1), which results in a greater surface area available for SP adsorption, and consequently, less free SP to improve the workability of the cement paste [10]. Studies evaluating the rheological properties of cementitious composites with CNT are scarce [51,53,54]. One of the few studies that show the detailed rheological characterization of CNT-reinforced cementitious pastes is that from Andrade Neto et al. [53], which also found a progressive increase in yield stress with the increase of CNT up to 0.10%. Additionally, the increase in yield stress caused by the incorporation of other nanomaterials has already been reported in the literature [55,56,57]. Mendoza Reales et al. [56] observed that incorporating 1% NS in cement paste increased its static τ_0_ from 30 to 260 Pa. Hou et al. [57] observed that the incorporation of 2.25% of colloidal NS increased the yield stress of paste from about 49 to 71 Pa (i.e., by 45%). Andrade et al. [58] reported that cement paste with 3% NS required 6 times more SP than plain cement paste did to reach a given sample spread related to the yield stress. In general, these authors attributed the yield stress increases to the extremely high specific surface area of the nanomaterials. 

#### 3.2.2. Isothermal Calorimetry

**Figure 9:**
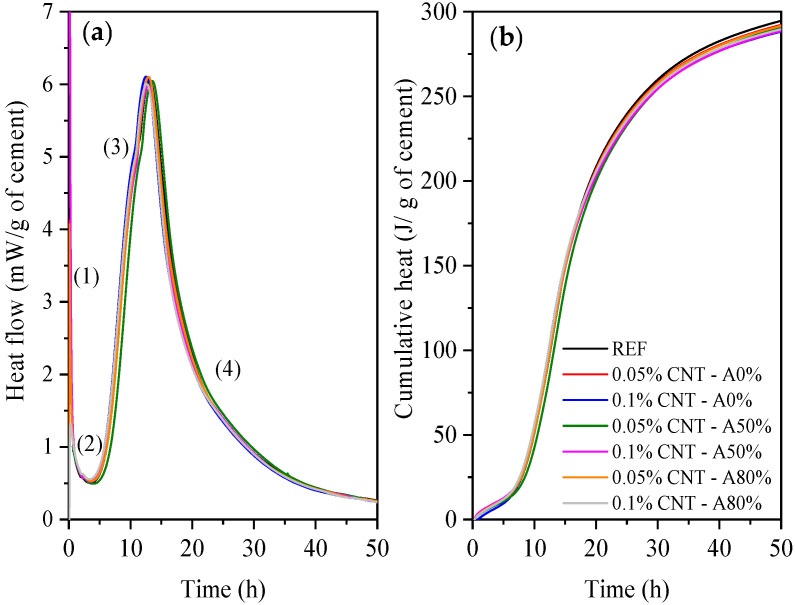
Heat flow (**a**) and cumulative heat (**b**) curves of cement pastes.

The heat flow and cumulative heat results of cement pastes are presented in Figure 9. Table 5 summarizes isothermal calorimetry results. Four steps were identified in the heat flow curves: (1) initial period; (2) induction period; (3) accelerating period; and (4) decelerating period. The initial period is related with the dissolution of cement phases. In the induction period, the initially rapid rate of reaction decreases down to remain a low rate, and mainly ionic chances occur. In the accelerating period, the precipitation of calcium silicate hydrates (C–S–H) and Ca(OH)_2_ occurs. Finally, the decelerating period is associated with the gradual reduction of the reaction rate [59]. The isothermal calorimetry results also indicated that the conversion of ettringite to AFm phases did not occur for up to 50 h. Conversion can be identified by a shoulder peak in the heat flow curve [60]. According to Bullard et al. [60], the AFm formation usually occurs after 50 h for cement with a 3.5% SO_3_, which is a usual C_3_A-to-sulfate ratio in modern cement. The isothermal calorimetry results are consistent with the XRD patterns of the cement pastes with 1 day of hydration further discussed, which also did not indicate the presence of AFm phases. 

According to Macleod et al. [61], the hydration kinetics of CNT nanocomposites by isothermal calorimetry has not been widely investigated. As pointed out by Andrade Neto et al. [53], the results regarding the effect of CNT on cement hydration are divergent. In general, CNT accelerates cement hydration [62,63]. In turn, some studies have reported hydration delays [64,65], which are usually attributed to either the material used for CNT dispersion like surfactant or SP admixtures [53], or else to an inefficient dispersion. 

CNT incorporation did not significantly change the main heat flow peak, although slightly anticipated its occurrence (by up to 4%), except for 0.05% CNT—A50%. The CNT used were functionalized with carboxyl groups and can absorb mixing water and reduce the effective w/c ratio due to their hydrophilic behavior [66,67], which results in increasing the concentration of alkaline ions, contributing to the acceleration of initial hydration reactions [68]. Nonetheless, all these variations were of up to 0.63 h compared with control pastes (REF).

It is stressed that the standard deviation associated with isothermal calorimetry repeatability is up to 7 J/g [68]. Thus, the 24 h and 48 h cumulative heat of all cement pastes evaluated are within this range and, in this way, can be attributed to the variability of the isothermal calorimetry test. Therefore, the obtained results indicated that the CNT incorporation did not promote significant changes in the hydration kinetics of OPC.

#### 3.2.3. Compressive Strength

**Figure 10:**
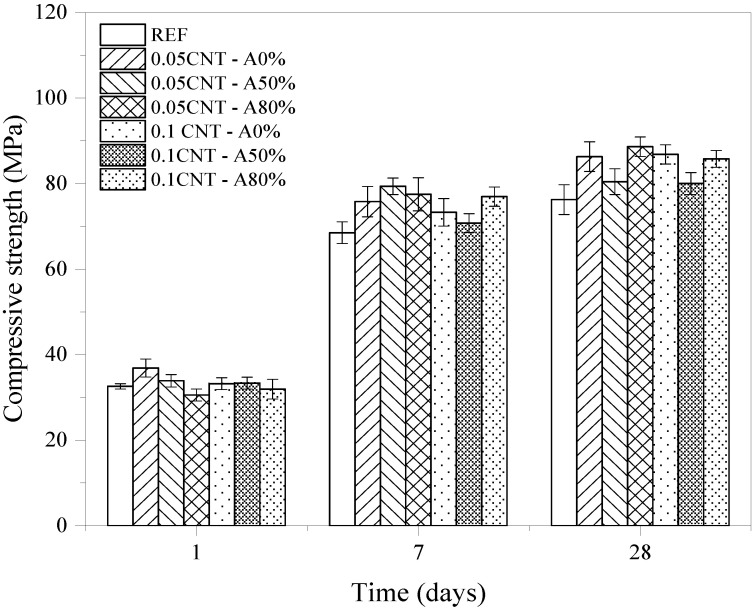
Compressive strength at 1, 7 and 28 days of the cementitious composites.

The average compressive strength values of the cement pastes are presented in Figure 10. Table 6 summarizes the ANOVA results of compressive strength values. The tree factors evaluated (CNT content, amplitude, and hydration time) had a significant influence on the compressive strength, as well as all the interactions between the factors, except for A × B. At 1 day, 0.05CNT—A0% presented a 13.2% increase in compressive strength compared with the plain cement paste (REF). The higher improvements with CNT incorporation were obtained at 7 days. In relation to the plain cement paste (REF), these increases were of 10.6% (0.05CNT—A0%), 15.8% (0.05CNT—A50%), and 13.1% (0.05CNT—A80%). At 28 days, these values were 13.8%, 4.9% and 12.5%, respectively. 

To complement the analysis of compressive strength results, Tukey’s multiple comparison test was performed. This test was used because is widely applied and allows to determine if there is a difference between the mean of all pairs of the study [69]. The results are shown in Table 7. The optimum CNT content was 0.05% by cement weight since the addition of 0.1% CNT generally led to lower compressive results. This content is close to the optimum level of 0.075% reported by Andrade Neto et al. [53]. Additionally, there was no significant difference between the amplitudes A0% and A80% results, while the comparisons between A0% and A50%, A50% and A80% had significant differences. These results indicate that, in general, the use of an amplitude of 50% in the ultrasonication process for CNT dispersion is not effective to improve the compressive strength results of cement pastes. In addition, since there are no significant statistic differences between A0% and A80%, it is concluded that the addition of CNT without ultrasonication in cement pastes was the best option from the point of view of compressive strength. Indeed, it led to the same mechanical performance as the pastes with CNT dispersed by ultrasonication with amplitude of 80%, without the dispersion step of CNT for incorporation in cementitious composites. It is worth highlighting that the unsonicated mixes (A0%) also had the best rheological performance among the CNT-containing mixes, reinforcing that ultrasonication may be avoided in functionalized CNT. This is because unsonicated mixed presented a smaller increase in the yield stress and viscosity of the cement pastes. This can be beneficial, since significant increases in yield stress and viscosity can reduce the flowability of cementitious composites. This can make it difficult for air bubbles to escape from the fresh mix, consequently increasing the porosity of the matrix [70]. 

Increases in compressive strength of cement pastes with CNT incorporation can generally be explained by the heterogeneous nucleation effect [39,53]. Additionally, the CNT bridging effect of hydrates and nano-cracks can provide mechanical reinforcement [71]. Similarly, for Chen and Akono [72], CNT enhanced the mechanical properties of cement pastes by high-density C–S–H and Ca(OH)_2_ formation, reducing the porosity of the matrix. 

Regarding the application of ultrasonication amplitudes of 50% and 80% for CNT dispersion, the energy provided may damage CNT and SP molecules, as previously discussed. Consequently, CNT may improve the mechanical performance of composite only by filling effect, while the nano-reinforcement (i.e., bridging effect) is no longer efficient due to CNT deterioration. According to Alatawna et al. [73], the ultrasonication of functionalized CNT promotes the shortening of CNT since nanotubes tend to break in defects induced by functional groups on the surface of the nanomaterial. Thus, the shorter CNT are less effective in enhancing the mechanical properties of composites. 

#### 3.2.4. Microstructure

**Figure 11:**
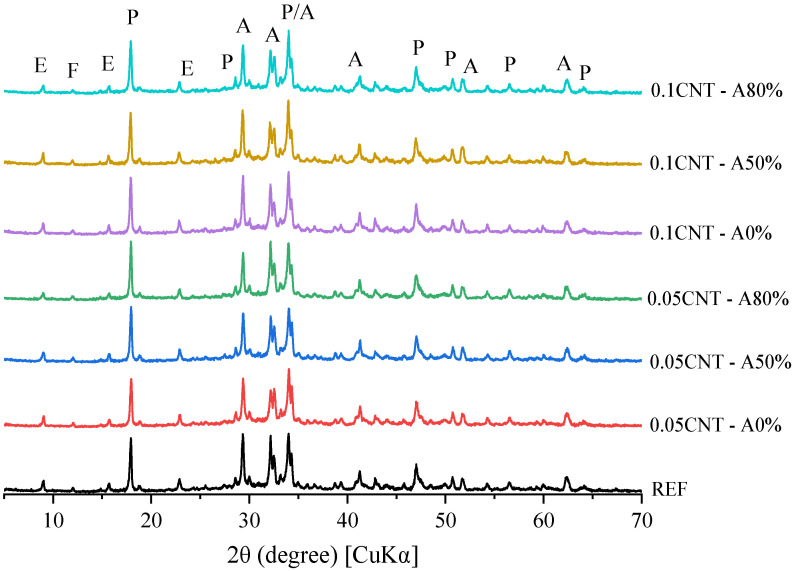
XRD patterns of cement pastes at 1 day of hydration (A—alite; E—ettringite; F—ferrite; P—portlandite).

**Figure 12:**
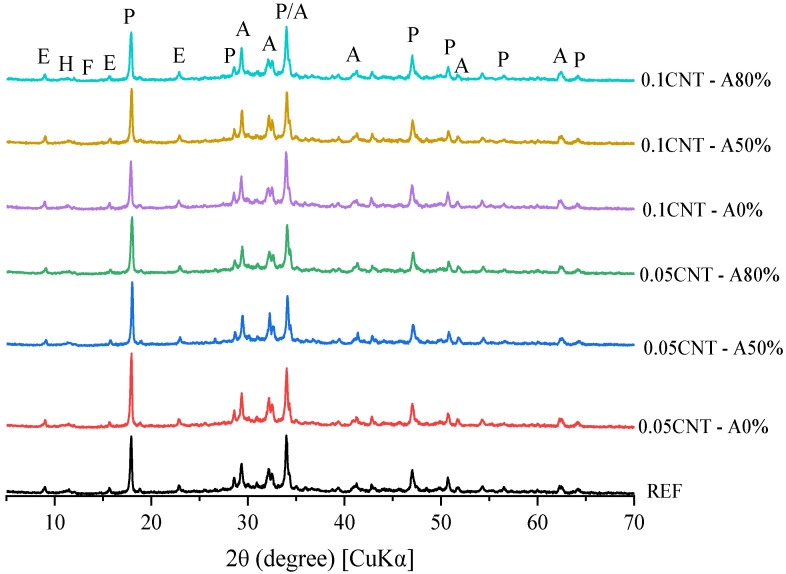
XRD patterns of cement pastes at 7 days of hydration (A—alite; E—ettringite; F—ferrite; H—hemicarbonate; P—portlandite).

**Figure 13:**
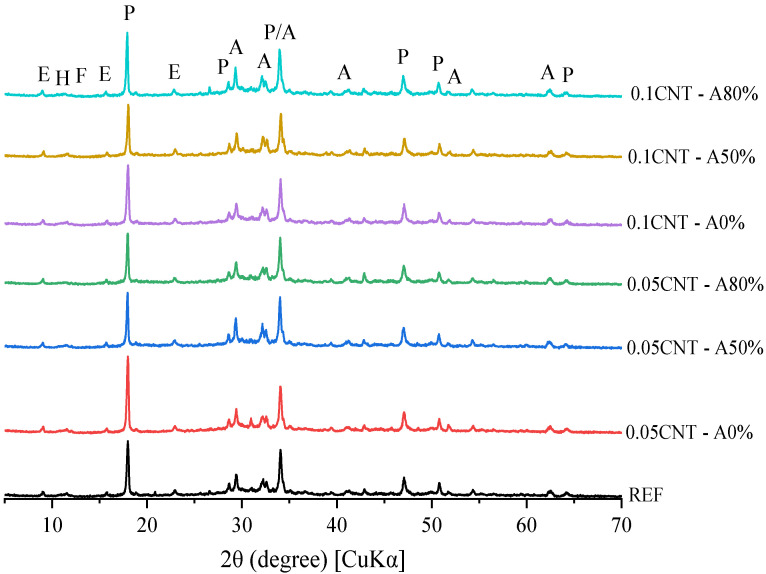
XRD patterns of cement pastes at 28 days of hydration (A—alite; E—ettringite; F—ferrite; H—hemicarbonate; P—portlandite).

**Figure 14:**
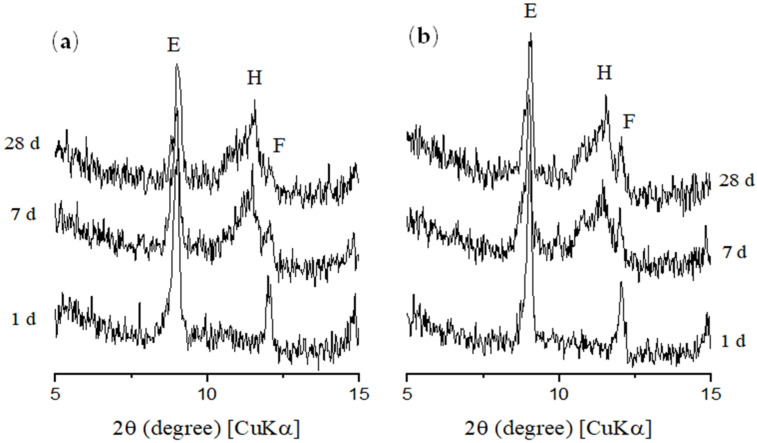
XRD patterns of (**a**) REF and (**b**) 0.05 CNT—A0% at 1, 7 and 28 days (E—ettringite; F—ferrite; H—hemicarbonate).

Figure 11, Figure 12 and Figure 13 show the XRD patterns of hydrated cement pastes at 1, 7 and 28 curing days, respectively. At 1 day of hydration, the main crystalline phases found were those from anhydrous cement (alite, ferrite, calcite, and periclase), in addition to portlandite and ettringite formed by the hydration reactions. A diffuse hump around 25–40° 2θ can already be found at this age, which corresponds to the amorphous (or nano-crystalline) contribution of C–S–H [29]. No AFm phases were found at this age, since the only reflection found up to 2θ = 12° was that associated with ettringite (at around 9.0° 2θ). This corroborates with the isothermal calorimetry results, which showed the absence of the typical broad peak of heat release at the deceleration period related with the formation of AFm up to 50 h. At 7 days, a considerable decrease in alite peaks (mainly at 2θ = 29.3, 32.2, 32.5, and 34.3°) were observed for all the samples, and this is associated with the progress in cement hydration. At this age, a broad peak at 2θ = 11.4 ° was observed, associated with the formation of Afm phases [74] due to the presence of calcite in the cement used, as exemplified in Figure 14 and confirmed latter by the TGA results. The same overall trend was observed in the XRD patterns of the samples with 28 days of hydration.

The XRD patterns of CNT-reinforced pastes were equivalent to that of plain cement paste (REF) at the respective hydration times, regardless of the CNT content and dispersion i.e., with and without ultrasonication. This indicates that that no new phases were formed due to the presence of CNT in the matrix during cement hydration as expected. This also indicates that the incorporation of CNT, either ultrasonicated or not, did not result in significant differences in cement hydration. Even though the extremely high surface area of CNT can provide extra surface for the nucleation and growth of hydrated products and therefore enhance the cement hydration [75], this nanomaterial was added at up to 0.1% by cement weight. This content may not be enough to result in significant changes in hydration. Similar results were reported by Andrade Neto et al. [53], who added up to 0.10% CNT in cement paste and found equivalent hydration degrees at 28 days, regardless of the CNT presence and content. One can note differences in the 001 reflection of portlandite (at 18.0° 2θ) when comparing samples at a given age e.g., 0.05% CNT—A0% vs. 0.1CNT—A0% at 7 days, and therefore expect different hydration degrees. However, the 101 reflection of portlandite at 2θ = 34.1° was equivalent for all the mixes at a given age, confirming the similar hydration degrees of the samples. This is explained by the flat-like hexagonal shape of portlandite crystals, which can face severe preferred orientation during sample preparation in powder XRD. This is discussed in detail in [76]. 

Finally, these results suggest that the compressive strength improvements promoted by CNT incorporation (seen in Section 3.2.3) were essentially mechanical, i.e., the so-called bridging effect, rather than the further formation of hydrated products.

**Figure 15:**
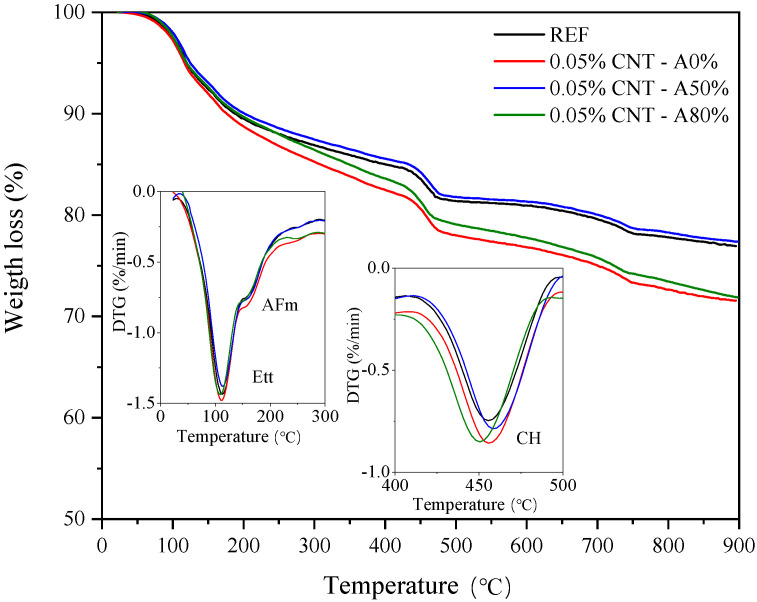
TGA and DTG curves of the pastes at 28 days of hydration. Ett: ettringite; AFm: aluminate phases; CH: calcium hydroxide.

Figure 15 shows the TGA curves of the pastes at 28 days of hydration and the insets show the DTG of the curves at specific regions. The large peak at 50–200 °C in DTG is associated with ettringite decomposition (pointed as Ett). A shoulder peak was also identified in this region at 150–200 °C, which is associated with the decomposition of aluminate phases (pointed as AFm), in line with the XRD results at this age. It is stressed that the hydration was stopped through solvent exchange (see Section 2.4), so no free water was found in TGA. Finally, the peak at 400–550 °C is assigned to the portlandite (i.e., Ca(OH)_2_) decomposition. Slight vertical displacements were observed in this region for the 0.05% CNT—A0% and —A80% DTG curves compared with the plain cement paste (REF) and CNT—A50% curves, which can be associated with the baseline displacement. Nonetheless, the relative height of the decomposition peaks was similar for all the samples.

Regarding the incorporation of CNT, DTG curves indicated equivalent contents of ettringite, monocarbonate and portlandite for all the pastes, at 28 days of hydration. These results suggest that CNT did not significantly influence the cement hydration at this age, which in line with the XRD results and corroborates with the hypothesis that CNT improved the mechanical strength of the composites essentially by mechanical reinforcement. 

## 4. Conclusions

This work investigated the influence of ultrasonication of functionalized carbon nanotubes (CNT) with different amplitudes on the rheology, hydration kinetics, compressive strength, and microstructure of Portland cement pastes. The increase in ultrasonication energy promoted a better dispersion of CNT in aqueous solution, evidenced by UV-Vis spectroscopy and DLS results. However, FTIR results from CNT plus superplasticizer (SP) solutions indicated that ultrasonication may have damaged both SP molecules and CNT. Nevertheless, additional characterization is needed to fully understand this phenomenon. 

Rheological tests evidenced that the increase in CNT content and ultrasonication amplitude gradually increased the static and dynamic yield stress, while decreased the mini slump spread of the paste. The higher surface area available in the ultrasonicated CNT systems can explain this trend.

CNT incorporation did not promote significant changes in the hydration kinetics of cement up to 50 h, with comparable heat flow and cumulative heat curves regardless of the CNT presence, content and ultrasonication.

Compressive strength results indicated that the optimum CNT content was 0.05% by cement weight, leading to strength increases of up to 15.8% compared with plain paste. The dispersion of CNT by ultrasonication with amplitudes of 50% and 80% did not contribute to increasing the compressive strengths compared with CNT without ultrasonication. 

XRD and TGA results indicated that CNT incorporation did not significantly affect the formation of hydrated products regardless of the CNT content and ultrasonication energy, corroborating the calorimetry results. These results suggest that the compressive strength improvements promoted by CNT were essentially mechanical.

Overall, ultrasonication of functionalized CNT is not efficient in improving the fresh and hardened performance of cementitious composites. In this context, the contribution of this work is extremely relevant from the point of view of the practical application of the functionalized CNT. This is because the results indicated that this type of CNT does not need to be dispersed by ultrasonication to improve the mechanical performance of cementitious composites. This is interesting since a limiting factor for the large-scale application of nanomaterials is precisely related to this dispersion step. 

## Figures and Tables

**Table 1:** CNT properties.

Inside Diameter (nm)	Outside Diameter (nm)	Length (µm)	SSA (m^2^/g)	Purity	−COOH Content (%)
5–10	20–30	10–30	>200	95%	1.9–2.1%

**Table 2:** Chemical composition of Portland cement.

Composition	SiO_2_	Al_2_O_3_	Fe_2_O_3_	CaO	K_2_O ^a^	MgO	SO_3_	LoI ^b^	IR ^c^
wt.(%)	18.31	4.46	2.84	60.76	0.66	3.37	3.08	3.79	0.72

^a^ Na_2_O equivalent, ^b^ Loss on ignition, ^c^ Insoluble residue.

**Table 3:** Composition of CNT aqueous dispersions.

CNT Dispersions	CNT (g)	Water (g)	SP (g)	Amplitude (%)	Energy (J)	Energy (J/mL)
0.05% CNT—A0%	0.05	40.00	0.20	-	-	-
0.05% CNT—A50%	0.05	40.00	0.20	50	9356	234
0.05% CNT—A80%	0.05	40.00	0.20	80	24,988	628
0.1% CNT—A0%	0.10	40.00	0.20	-	-	-
0.1% CNT—A50%	0.10	40.00	0.20	50	9361	234
0.1% CNT—A80%	0.10	40.00	0.20	80	24,776	620

**Table 4:** Average particle size and polydispersity index (PDI) of CNT dispersions.

CNT Dispersions	Average Particle Size (nm)	PDI
0.05% CNT—A50%	158.1	0.381
0.05% CNT—A80%	138.0	0.322
0.1% CNT—A50%	143.2	0.355
0.1% CNT—A80%	130.1	0.275

**Table 5:** Isothermal calorimetry results.

Cement Paste	Main Heat Flow Peak (mW/g)	Main Heat Flow Peak Time (hh:mm)	24 h Cumulative Heat (J/g)	48 h Cumulative Heat (J/g)
REF	6.05	13:08	234.4	292.6
0.05% CNT—A0%	6.08	12:57	232.9	290.3
0.1% CNT—A0%	6.11	12:30	230.5	286.4
0.05% CNT—A50%	6.05	13:30	227.5	288.5
0.1% CNT—A50%	5.98	12:50	228.7	286.7
0.05% CNT—A80%	6.10	12:54	232.3	289.7
0.1% CNT—A80%	6.00	12:36	230.9	287.8

**Table 6:** Three-way ANOVA of compressive strength results.

Factor	Sum of Squares (SS)	Degrees of Freedom (DF)	Mean Square (MS)	*p* Value	Sig. ^a^
CNT content (A)	157.249	1	157.249	5.84 × 10^−7^	S
Amplitude (B)	203.390	2	101.695	1.74 × 10^−7^	S
Time (C)	45,132.668	2	22,566.334	0	S
A × B	13.668	2	6.834	0.27857	NS
A × C	121.266	2	60.633	4.35 × 10^−5^	S
B × C	418.700	4	104.675	3.65 × 10^−11^	S
A × B × C	106.011	4	26.503	0.00119	S
Error	388.891	74	5.255	-	-
Total	46,541.843	91	-	-	-

^a^ S—Significant, NS—Not significant.

**Table 7:** Tukey’s multiple comparison test of compressive strength results.

Factor	Levels	Mean Difference	Standard Error of the Mean (SEM)	q Value	Prob.	Sig. ^a^
CNT content	0.05–0.1%	2.825	0.463	8.625	8.52 × 10^−8^	S
Amplitude	A0–A50%	2.670	0.521	7.236	7.00 × 10^−6^	S
Amplitude	A0–A80%	1.008	0.599	2.378	0.219	NS
Amplitude	A50–A80%	3.679	0.577	9.004	1.95 × 10^−8^	S

^a^ S—Significant, NS—Not significant.

## Data Availability

Not applicable.

## Abbreviations

CNT	carbon nanotubes
SP	superplasticizer
DLS	dynamic light scattering
FTIR	Fourier transform infrared spectroscopy
XRD	X-ray diffraction
TGA	thermogravimetric analysis
TEM	transmission electron microscopy
PDI	polydispersity index
